# Effects of Aerobic Exercise on Serum Adiponectin Concentrations in Children and Adolescents with Obesity: A Systematic Review and Meta-Analysis

**DOI:** 10.3390/life13081772

**Published:** 2023-08-18

**Authors:** Yunqing Zhang, Yigao Wu, Xueyin Fei, Yixuan Li, Yanchun Li, Xu Yan

**Affiliations:** 1 China Basketball College, Beijing Sport University, Beijing 100084, China; zhangyunqing325429@163.com; 2 China Institute of Sports and Health Science, Beijing Sport University, Beijing 100084, China; wuyigao521@gmail.com (Y.W.); 2022241088@bsu.edu.cn (Y.L.); 3 Sport Science School, Beijing Sport University, Beijing 100084, China; 1004320180587@bsu.edu.cn; 4 Institute for Health and Sport, Victoria University, Melbourne 3011, Australia; 5 Australian Institute for Musculoskeletal Science, Melbourne 3021, Australia; 6 Department of Medicine-Western Health, The University of Melbourne, Melbourne 3021, Australia

**Keywords:** aerobic exercise, adiponectin, obesity, children and adolescents

## Abstract

Serum adiponectin plays a vital role in various physiological processes, such as anti-inflammatory, anti-atherosclerotic, anti-apoptotic and pro-angiogenic activities. Any abnormalities in its concentration can lead to adverse health outcomes, particularly in children and adolescents. Therefore, it is crucial to investigate factors influencing serum adiponectin concentrations in this population. The primary objective of this study was to systematically evaluate the impact of aerobic exercise on serum adiponectin concentrations in children and adolescents with obesity. To achieve this, a comprehensive literature search was conducted up to January 2023, utilising five databases: PubMed, Web of Science, Embase, Cochrane Library and Clinicaltrial.gov. The inclusion criteria involved studies that focused solely on aerobic exercise as an intervention for children and adolescents with obesity. Only studies that reported outcome indicators related to serum adiponectin were considered for analysis. The quality of the included studies was assessed using the Cochrane Risk of Bias (ROB) assessment tool, and statistical analysis was performed using RevMan 5.4.1 analysis software. This meta-analysis incorporated data from eight trials, involving a total of 272 subjects. The results demonstrated that aerobic training significantly increased serum adiponectin concentrations [standardized mean difference (SMD) = 0.85; 95% confidence interval (CI) = 0.33 to 1.37; I^2^ = 0%; *p* = 0.001] in children and adolescents with obesity when compared to non-exercise controls. Furthermore, the magnitude of this effect appears to be influenced by the intensity of aerobic exercise, with higher-intensity aerobic exercise resulting in greater increases in serum adiponectin concentrations.

## 1. Introduction

In recent years, childhood and adolescent obesity has emerged as a critical global health concern. According to data from the World Health Organization (WHO) and the World Obesity Federation (WOF), the number of children and adolescents with obesity between 5 and 19 years of age exceeded 150 million worldwide in 2020 [1]; this number is projected to surpass 250 million by 2030 [1]. The rising rates of obesity in this age group bring a range of potential complications. Some of the main complications associated with obesity in children and adolescents include an increased risk of chronic diseases such as type 2 diabetes, atherosclerosis and other cardiovascular diseases [2]; a higher likelihood of obesity persisting into adulthood [3,4]; elevated mortality and premature death risk [5]; and abnormalities in hormone levels, including serum adiponectin [6]. Notably, abnormal serum adiponectin concentrations have strong associations with obesity, type 2 diabetes, cardiovascular disease and certain cancers [7]. These associations may be linked to various effects of serum adiponectin, such as its anti-inflammatory, anti-atherosclerotic, anti-apoptotic, pro-angiogenic and insulin-sensitizing properties [7]. Previous studies have demonstrated that changes in physical activity [8] can affect serum adiponectin concentrations.

Physical activity is a crucial component of normal growth and overall health for children and adolescents [9]. According to the 2020 WHO Guidelines on Physical Activity and Sedentary Behavior for Children and Adolescents 5–17 years of age, it is recommended that children and adolescents engage in at least 60 min of moderate-intensity aerobic exercise daily [10]. Aerobic exercise is a widely adopted form of physical activity with numerous benefits, such as improving body shape and cardiorespiratory fitness in children and adolescents [11]. Additionally, aerobic exercise has been shown to play a preventive and therapeutic role in various chronic diseases, and it can improve abnormal levels of triglycerides, total cholesterol, high-density lipoprotein cholesterol and low-density lipoprotein cholesterol in the body.

While many studies have explored the effects of aerobic exercise on serum adiponectin concentrations in various populations [12,13,14,15,16], there is a scarcity of research specifically focusing on children and adolescents with obesity. Previous studies have indicated that aerobic exercise can increase the concentration of serum adiponectin in individuals with obesity and improve its abnormal secretion [12,13,14,15]. However, its specific impact on serum adiponectin levels in children and adolescents with obesity remains unclear. Therefore, we conducted a systematic review and meta-analysis, analysing relevant randomised controlled trials, in order to assess the effect of aerobic exercise on serum adiponectin levels in children and adolescents with obesity. This study aims to contribute valuable insights to the prevention and treatment of various chronic diseases in children and adolescents, as well as to the regulation of abnormal adiponectin secretions.

## 2. Materials and Methods

### 2.1. Protocol

This study adhered strictly to the guidelines for systematic evaluation and meta-analysis (PRISMA) [17]. In defining the inclusion criteria, we followed the PICOS model [18] (see Table 1). Additionally, this study has been registered on the International Prospective Register of Systematic Evaluations (PROSPERO) website under the registration number CRD42023390011.

### 2.2. Literature Sources and Search Strategies

All of the literature included in this systematic evaluation was sourced from five databases: PubMed, Web of Science, Embase, Cochrane Library and Clinicaltrial.gov. The literature search was conducted up until January 2023, encompassing publications that met the criteria from the above-mentioned databases up to that date.

The search strategy was developed independently by the author of this paper (Yunqing Zhang) with the guidance of a professor (Yanchun Li), who has been trained by medical librarians. To ensure accuracy and reliability, the search strategy was reviewed and checked by two peer reviewers (Yigao Wu and Xueyin Fei) using the structured peer review tool (The PRESS 2015 Guideline Statement) [19]. Firstly, the author filled out the pertinent information in the PRESS 2015 Guideline Assessment Form for the search strategy. After that, the completed form was sent to the peer reviewers. The peer reviewers used the PRESS 2015 Evidence-Based Checklist, and reviewed the search strategy according to the criteria in the table [19]. Once the search strategy was confirmed to be appropriate, manual searches were carried out in the five databases by Yunqing Zhang. The search terms used were “Adiponectin”, “child” and “adolescent”, while the free terms used were “Aerobic Exercise”, “Exercise, Aerobic”, “apM-1 Protein”, “ACRP30 Protein”, “Children” and “Adolescents”. The Boolean operators OR and AND were used to develop the search form and refine the results. In PubMed, a combination of subject terms (MeSH) and free terms was utilised for the searches. The complete search strategy used in the PubMed database is shown in Table 2.

In the final stage of the literature selection, two independent evaluators (Yixuan Li and Guihua Sun) carefully reviewed the titles and abstracts of all retrieved papers. They compared each paper against the inclusion and exclusion criteria to conduct the initial screening. Papers that met all of the inclusion criteria were subjected to a second screening, which involved reading the full text of each selected paper. In cases where there were disagreements during the screening process between the two evaluators, they engaged in discussions to resolve the discrepancies. However, if they could not reach a consensus, a third evaluator (Yigao Wu) was consulted to provide additional input and facilitate resolution of the matter. Through a series of screening exercises and thoughtful discussions, the final selection of literature that met all of the inclusion criteria was determined.

### 2.3. Criteria for Inclusion and Exclusion from the Literature

The following criteria were used to evaluate the inclusion of literature in this paper: (1) the participant population consisted of children and adolescents with a mean age between 5 and 19 years [20]; (2) the intervention in the experimental group focused solely on aerobic exercise, and did not include other types of physical exercises such as resistance training; (3) the control group was a blank control with no exercise intervention; (4) the outcome indicator had to include adiponectin; (5) the randomised controlled trial (RCT) was published in English.

The following criteria were used to evaluate the exclusion of literature in this paper: (1) the average of the intervention population was less than 5 years or greater than 19 years; (2) literature where the full text was not available or where the outcome indicator did not include adiponectin; (3) literature in the format of conference abstracts, case reports, guidelines, letters, reviews, etc.; (4) studies conducted on animals; (5) interventions in the experimental group that were not solely aerobic, and involved combined exercise or interventions in the control group with exercise interventions.

### 2.4. Study Selection and Data Extraction

Firstly, the two evaluators (Yixuan Li and Guihua Sun) imported the literature retrieved from the five databases into the literature management software (EndNote 20) and removed any duplicates using the software. Based on the titles and abstracts of the literature, the evaluators excluded all studies that did not meet the criteria. The remaining literature underwent a thorough evaluation by the two evaluators, who read the full text of each paper to ensure it met all the inclusion. Next, the two evaluators (Yixuan Li and Guihua Sun) independently extracted data from the selected studies.

It is important to note that during the study selection process, a study would be excluded if it did not report sufficient data (e.g., no mean (mean) ± standard deviation (SD) or appropriate effect size data). If a study included multiple interventions (e.g., different intensities, frequencies, durations of aerobic exercise, or different groups of participants in the age range) in which multiple intervention groups met the inclusion criteria, these interventions were treated as separate randomised controlled trials for inclusion in the meta-analysis.

A data extraction form, designed by the author of this paper (Yunqing Zhang), was used by the two evaluators to capture the required information including the following: (1) study characteristics (name of first author, year of publication, journal of publication, region of study and weight status of the experimental population); (2) participant characteristics (sample size, age and gender); (3) experimental design (type of experiment, duration, intensity and frequency of intervention); (4) outcome indicators (serum adiponectin). The data extracted by the evaluators were cross-checked with each other to ensure accuracy and consistency. If any disagreement arose, a third evaluator (Yigao Wu), reviewed, discussed and resolved the discrepancies. In addition, the author of this paper (Yunqing Zhang) reached out to the authors of the included literature that lacked sufficient data; an email was sent to these authors, requesting the missing information.

### 2.5. Quality Assessment

In this systematic evaluation, the quality of the included literature was assessed using the Cochrane Risk of Bias (ROB) tool [21]. Two evaluators (Yixuan Li and Guihua Sun) independently evaluated each study to identify potential sources of bias, including selectivity bias, implementation bias, follow-up bias, reporting bias and other biases. The results were categorised into three criteria: low risk of bias, high risk of bias and unclear [22]. If there were insufficient data in a study to make a reasonable judgment, the domain was classified as having an unclear risk of bias. In case of any disagreements between the two evaluators (Yixuan Li and Guihua Sun), a third evaluator (Yigao Wu) was consulted to discuss and reach a consensus. Finally, the assessment of the risk of bias for the included literature is visually presented in this study through Review Manager 5.4.1 software.

### 2.6. Data Analyses

This systematic evaluation employed statistical analysis according to the Cochrane Handbook for Statistical Reviews of Interventions [23]. The data were analysed using Review Manage 5.4.1 software (Cochrane, London, UK). The effect sizes (ES) were presented as standardised mean differences (SMD) with 95% CI calculated. Heterogeneity among the studies was evaluated using the consistency coefficients I^2^ and *p*-values [24]. If I^2^ was greater than 50% and the *p*-value was less than 0.10, heterogeneity was considered to exist; then, a random-effects model was used for the meta-analyses. Conversely, if there was no significant heterogeneity, a fixed-effects model was used. If there existed heterogeneity, subgroup analyses were conducted to explore potential sources of heterogeneity by comparing aerobic training duration, intensity and frequency. The results of this study indicated I^2^ = 0% and *p* = 0.44, suggesting no significant heterogeneity between studies; therefore, a fixed-effects model was used for the analyses.

A sensitivity analysis was performed to assess the influence of individual studies on the overall effect. Each study was systematically removed from the model once, and its impact on the overall results was evaluated. In cases where statistical heterogeneity existed without clinical heterogeneity being observed, a random-effects model was utilised for the analyses. When heterogeneity was apparent but its source could not be identified, only descriptive analyses were performed. To identify potential publication biases, funnel plots and Egger’s regression were employed. The significance level for the tests was set at α = 0.05. The Z-test was used to determine the statistical significance of the results, with a *p*-value below 0.05 indicating significance.

## 3. Results

### 3.1. Selection of Literature

**Figure 1:**
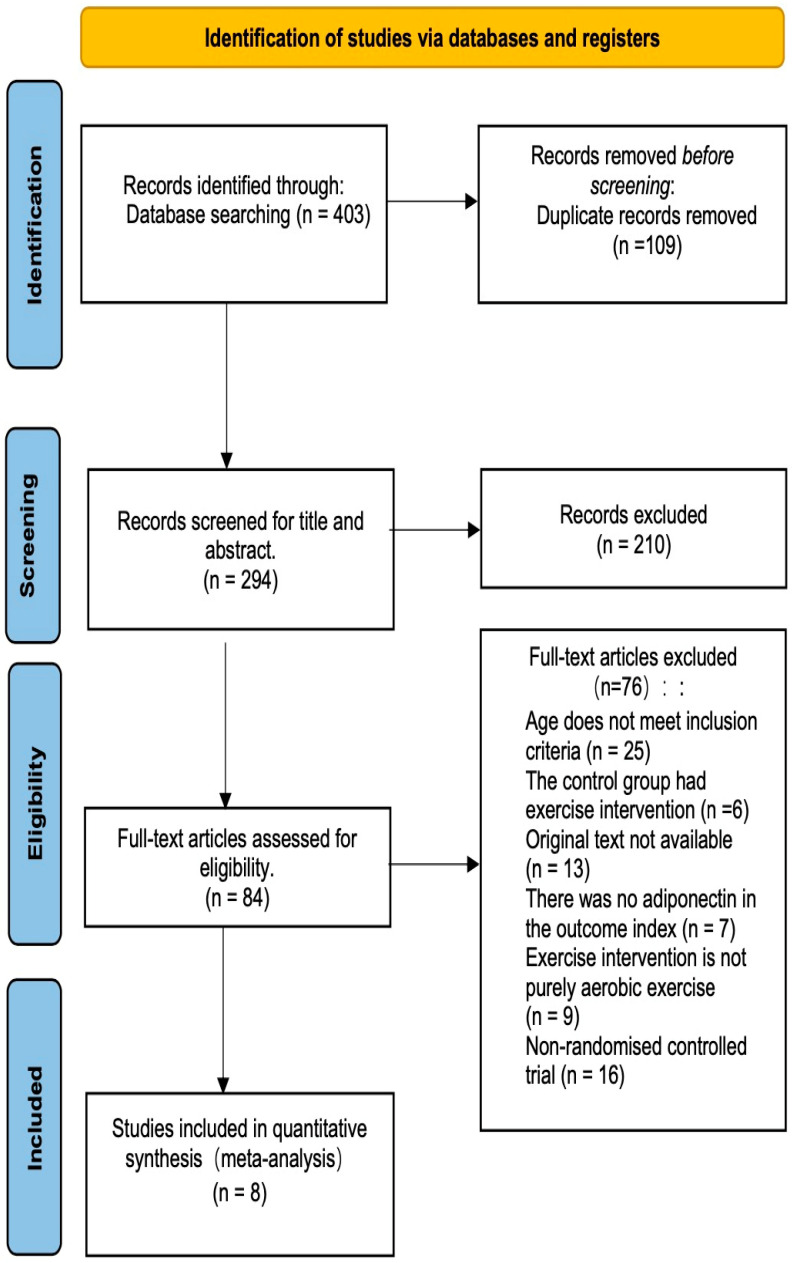
Flowchart of study selection for systematic review and meta-analysis.

This systematic evaluation commenced with a comprehensive search across five databases, yielding 403 documents. After removing 109 duplicate records, 210 documents underwent initial screening based on their titles and abstracts. Subsequently, 84 documents were selected for further evaluation, and their full texts were thoroughly reviewed. Consequently, 76 documents were excluded as they did not meet the inclusion criteria, resulting in 8 documents that met all of the inclusion criteria (See Figure 1) [25,26,27,28,29,30,31,32].

### 3.2. Quality Assessment of Studies

**Figure 2:**
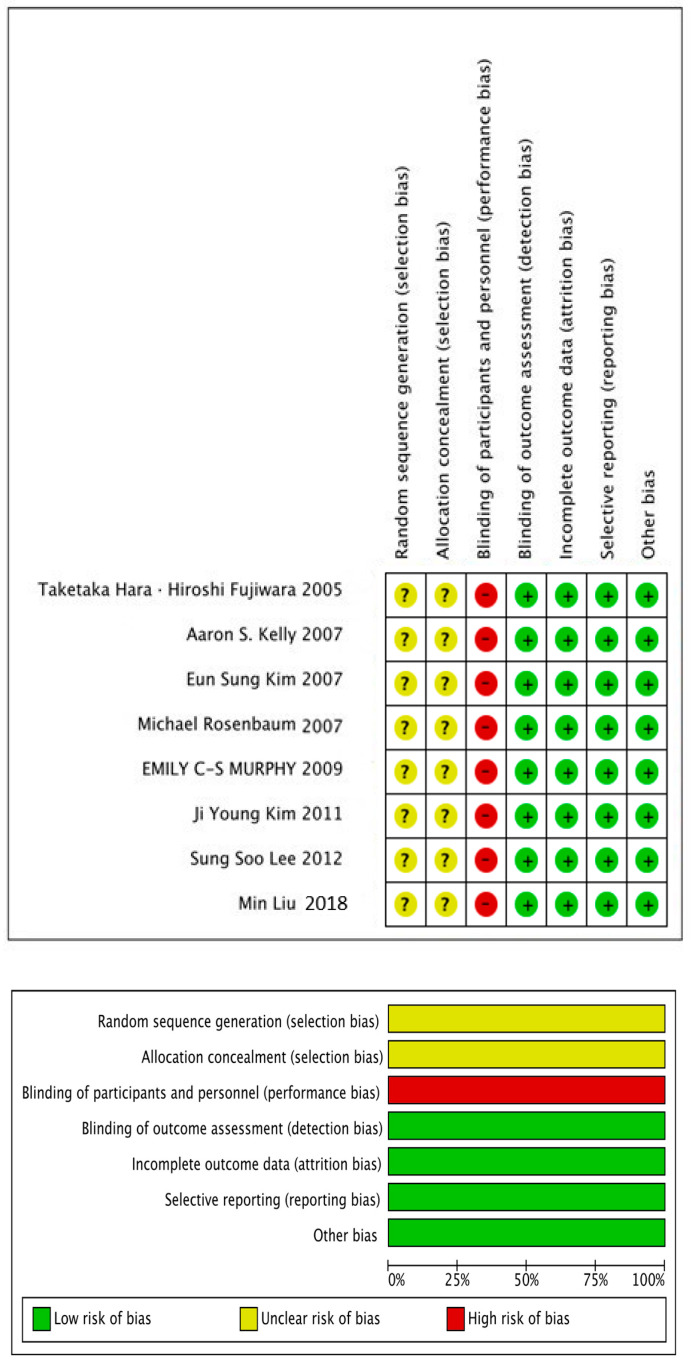
Risk of bias summary. Note: +—low risk of bias; −—high risk of bias; ?—bias [25,26,27,28,29,30,31,32].

The quality and risk of bias for the eight included publications were evaluated using the Cochrane Risk of Bias tool. The results of this assessment are presented in Figure 2.

### 3.3. Basic Characteristics of the Included Literature

The key characteristics of the included literature are presented in Table 3 [25,26,27,28,29,30,31,32]. This table provides essential information about the studies, including author names, countries of origin, publication years, participant age, sample sizes, details of exercise interventions and weight status. A total of eight randomized controlled trials (RCTs) were included in this systematic evaluation, encompassing a sample size of 272 participants. Geographically, three studies were conducted in the United States [26,28,29] and three in Korea [27,30,31], while there was one study each from China [32] and Japan [25]. All of the included RCT trials focused on participants with obesity.

In the experimental design section, children and adolescents with obesity were recruited using various methods, such as leaflets or school directories. Then, they were randomly assigned to a control group and a trial group for the exercise interventions. The trial group participated in different training programs based on the experimental design, all of which involved aerobic exercises. The control group, on the other hand, did not undergo any exercise intervention. The classification of aerobic exercise intensity in this meta-analysis followed the criteria established by the American College of Sports Medicine (ACSM) [33]. According to the ACSM criteria, aerobic exercise can be categorised into three intensity levels: high-intensity aerobic exercise [≥6 metabolic equivalents (METs), ≥70% HRmax, ≥60% heart rate reserve (HRR), or ≥60% maximal oxygen consumption (VO2max)]; moderate-intensity aerobic exercise (3–6 METs, 55–70% HRmax, 40–60% HRR, or 40–60% VO2max); and low-intensity aerobic exercise (<3 METs, <55% HRmax, <40% HRR, or <40% VO2max). This classification was used to categorise the aerobic exercise interventions included in this meta-analysis.

### 3.4. Meta-Analyses

**Figure 3:**
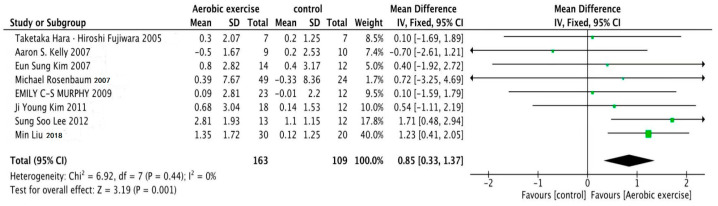
Meta-analyses of the effect of aerobic exercise on serum adiponectin concentrations in children and adolescents with obesity [25,26,27,28,29,30,31,32].

Figure 3 displays a forest plot illustrating the collective impact of aerobic exercise on serum adiponectin concentrations in children and adolescents with obesity. The analysis showed no heterogeneity among the studies (I^2^ = 0%, *p* = 0.44). The analyses implied a fixed effects model, yielding a combined effect size of SMD = 0.85, 95% CI = 0.33~1.37 (*p* = 0.001). These findings indicate that aerobic exercise has a significant positive effect on increasing serum adiponectin concentrations in children and adolescents with obesity.

Out of the eight studies included in these analyses, one study reported a decrease in serum adiponectin concentrations following the aerobic exercise intervention, while an increase was observed in the control group [26]. However, the remaining seven studies demonstrated that aerobic exercise, with various durations, intensities and frequencies, led to an increase in serum adiponectin concentrations in children and adolescents with obesity, albeit to varying degrees [25,27,28,29,30,31,32].

### 3.5. Publication Bias and Sensitivity Analyses

**Figure 4:**
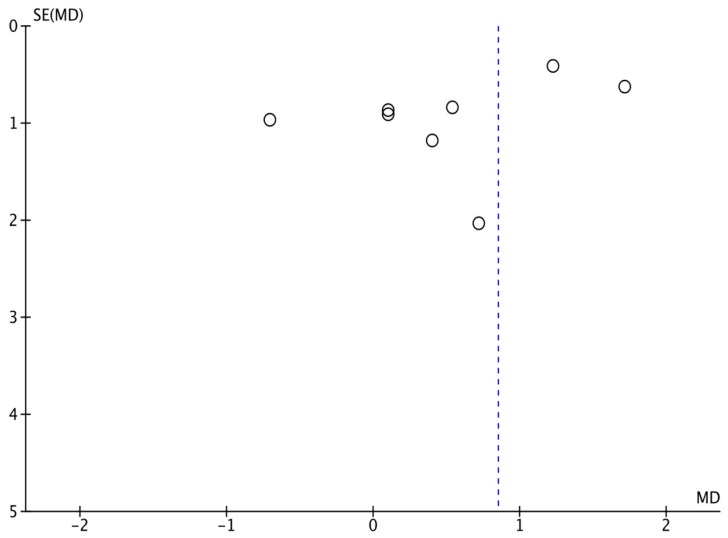
Publication bias in the effects of aerobic exercise on serum adiponectin concentrations in children and adolescents with obesity.

The funnel plots [24] depicting the impact of aerobic exercise on serum adiponectin concentrations in children and adolescents with obesity revealed an asymmetrical distribution, as illustrated in Figure 4. This observation suggests the presence of potential publication bias, although it is important to consider other factors that may contribute to this asymmetry. To further assess potential publication bias, Egger’s regression analysis was conducted, yielding a *p*-value of 0.001, indicating the existence of publication bias in this systematic evaluation.

To ensure the robustness of the results, sensitivity analyses were performed by manipulating the joint effect size and excluding individual study observations. The analysis involved removing specific studies prior to conducting the meta-analysis. The findings indicated that the alteration in the joint effect size, after excluding individual studies, was not statistically significant when compared to the previous joint effect size. These results demonstrate the stability of the meta-analysis, and provide confidence in the study’s conclusions.

## 4. Discussion

This study represents a pioneering effort to comprehensively assess the impact of aerobic exercise on serum adiponectin concentrations in children and adolescents with obesity. By incorporating eight trials with a total of 272 subjects, this systematic evaluation yielded noteworthy findings. Specifically, it was found that in the absence of concurrent dietary modification or other lifestyle adjustments, the exercise group exhibited a significant increase in serum adiponectin concentrations following aerobic exercise with various durations, intensities and frequencies. The combined effect size was SMD = 0.85 (95% CI = 0.33 to 1.37), providing strong support for a significant effect. This indicates notable health benefits of aerobic exercise for children and adolescents with obesity [25,27,28,29,30,31,32]. Importantly, the absence of heterogeneity among the studies, as confirmed by an I^2^ value of 0% and a *p*-value of 0.44, enhances confidence in the findings.

Although the precise mechanisms underlying the impact of aerobic exercise on serum adiponectin concentrations are not fully understood, ongoing research endeavours are dedicated to unraveling these intricacies. In line with this pursuit, one study [34] suggested that interleukin-6 (IL-6, interleukin) may play a role in modulating serum adiponectin mRNA expression. The findings indicate that individuals experiencing reduced IL-6 concentrations during exercise exhibit a significant increase in serum adiponectin mRNA, likely due to the diminished concentration of IL-6. This observation implies that aerobic exercise potentially influences serum adiponectin concentrations indirectly through a reduction in IL-6. Furthermore, it is worth noting that alterations in serum adiponectin concentrations may be closely associated with changes in certain anthropometric indicators, such as the BMI [35].

Several factors influence the extent of aerobic exercise to promote serum adiponectin secretion concentrations in children and adolescents with obesity. The effect of aerobic exercise on serum adiponectin has been associated with various factors, such as weight changes [36], exercise intensity [35,37] and exercise duration [35,38]. In a three-year randomised controlled trial, it was reported that weight loss in subjects with obesity was associated with an increase in serum adiponectin concentrations. Specifically, a weight loss of at least 10% was required to trigger an elevation in serum adiponectin concentrations, while no substantial change in serum adiponectin was observed if there was no change in weight [36]. An animal experiment demonstrated [36] that high-intensity exercise contributed to fat loss, and may stimulate adiponectin secretion. Another study [38] emphasised the significance of exercise duration in serum adiponectin concentrations. The study revealed differences in changes in serum adiponectin concentrations between short-term exercise (<12 weeks) and long-term exercise (≥12 weeks). Short-term exercise did not significantly impact serum adiponectin concentrations, whereas long-term exercise resulted in elevated serum adiponectin concentrations. Among the included randomised controlled trials, aerobic exercise interventions with higher intensity, greater frequency and longer training periods exhibited more pronounced changes in serum adiponectin concentrations. Conversely, interventions with lower intensities, frequencies and shorter training periods yielded smaller changes in serum adiponectin concentrations. These findings align with those of previous studies [35,37,39], which highlighted that higher-intensity, more frequent and longer durations of aerobic exercise could facilitate weight loss and increase serum adiponectin concentrations in children and adolescents with obesity. Although the precise mechanism behind this effect remains unclear, it is evident that prolonged, high-intensity and frequent aerobic exercise effectively reduces adiposity and increases serum adiponectin concentrations in children and adolescents with obesity.

Not all of the studies included in the analysis provided evidence supporting the notion that aerobic exercise increases serum adiponectin concentrations in children and adolescents with obesity. Specifically, one trial [26] reported that serum adiponectin concentrations did not increase in the exercise group of children and adolescents. In contrast, the control non-exercise group exhibited an increase in serum adiponectin concentrations in children and adolescents with obesity. The aerobic exercise intervention in this particular trial involved four sessions of stationary cycling per week for eight weeks. Initially, moderate-intensity exercise was implemented for the first two weeks, gradually progressing to high-intensity aerobic exercise. Interestingly, the exercise intensity in this particular study was not significantly lower compared to other similar interventions, yet the outcomes diverged from the anticipated findings [37]. This suggests that exercise intensity may not be the sole determinant influencing serum adiponectin concentrations. It also indicates that exercise is not the exclusive factor affecting serum adiponectin concentrations. When comparing before and after exercise, we found that the exercise group of children and adolescents did not lose weight. The lack of change in body weight before and after exercise is a potential explanation for the dissimilar results. It is plausible that dietary factors may have contributed to the absence of weight change, consequently impacting serum adiponectin concentrations. Thus, it is likely that the disparity between the results of this study and other studies is associated with body weight, dietary interventions or a combination of them, rather than solely exercise. This hypothesis is consistent with the results of previous studies on the effects of dietary or weight loss factors on serum adiponectin concentrations [40,41,42,43,44].

This study is subjected to several limitations. Firstly, the method of randomisation sequence was unclear in all of the included randomised controlled trials, introducing potential selective bias to this systematic evaluation. Secondly, it remains unclear from the available information whether allocation concealment was adequately reported in all the randomised controlled trials, which may introduce selective bias into this systematic evaluation. Thirdly, none of the randomised controlled trials included in this analysis reported blinding of outcome assessors, introducing the possibility of two implementation biases in the systematic evaluation. Furthermore, the presence of publication bias was identified through funnel plots and Egger’s regression, suggesting that studies with significant results may be more likely to be published. Another limitation is the relatively small sample size in some of the included randomised controlled trials, with fewer than 20 participants per group. Such small sample sizes may limit the statistical power of the trials and increase the risk of drawing inaccurate conclusions. Therefore, it is essential to conduct further studies with large sample sizes to validate and strengthen the current findings.

## 5. Conclusions

Aerobic exercise shows promise in increasing serum adiponectin concentrations in children and adolescents who are overweight and obese, offering a viable treatment option for addressing abnormal adiponectin concentrations in this population. The findings of this review provide evidence supporting the effectiveness of aerobic exercise in raising serum adiponectin concentrations in this population. Additionally, the impact of exercise on serum adiponectin concentrations is influenced by the intensity of the aerobic activity, with higher-intensity aerobic exercises tending to yield greater increases in serum adiponectin concentrations. However, it is important to note that the effectiveness of aerobic exercise in raising serum adiponectin concentrations is closely tied to its impact on weight loss in children and adolescents with obesity. If aerobic exercise does not facilitate weight loss in this population, the increase in serum adiponectin concentrations may be minimal or even absent. Therefore, when utilising aerobic exercise to improve serum adiponectin concentrations in children and adolescents with obesity, it is crucial to consider not only the intensity of the exercise but also its effect on weight reduction. A weight reduction is necessary to induce a significant increase in serum adiponectin concentrations in this population.

This systematic evaluation provides essential reference information for regulating abnormal adiponectin concentrations in children and adolescents with obesity through the implementation of aerobic exercise. This study holds the potential to inspire future researchers to expand upon existing knowledge, and conduct more comprehensive investigations into the effects of different forms of exercise on serum adiponectin concentrations and other markers of inflammation in this population. Further studies can explore a broader range of exercise modalities and delve into the specific effects of different types of exercise interventions, including variations in the periodicity, intensity and frequency, on serum adiponectin concentrations in children and adolescents with obesity. By examining these factors in greater detail, researchers can gain a deeper understanding of the most effective exercise approaches for modulating serum adiponectin concentrations in this specific population.

## Figures and Tables

**Table 1:** PICOS criteria for the inclusion of studies in the systematic review.

Parameter	Inclusion Criteria
Population	Children and adolescents
Intervention	Aerobic exercise
Comparators	No additional physical exercise
Outcomes	adiponectin
Study design	All randomised controlled trials

PICOS (population; intervention; comparators; outcomes; study design).

**Table 2:** Database retrieval strategy.

#1 “Adiponectin”[Mesh]
#2 (Adipocyte Complement-Related Protein 30-kDa [Title/Abstract])) OR (Adipocyte Complement-Related Protein 30 kDa [Title/Abstract])) OR (Adipose Most Abundant Gene Transcript 1 [Title/Abstract])) OR (apM-1 Protein [Title/Abstract])) OR (apM 1 Protein [Title/Abstract])) OR (ACRP30 Protein [Title/Abstract])) OR (Adipocyte, C1q [Title/Abstract] AND Collagen Domain-Containing Protein [Title/Abstract])
#3 #1 or #2
#4 (Aerobic Exercise [Title/Abstract]) OR (Exercise, Aerobic [Title/Abstract])) OR (Aerobic Exercises [Title/Abstract])) OR (Exercises, Aerobic [Title/Abstract])) OR (Aerobic Training [Title/Abstract])) OR (Training, Aerobic [Title/Abstract])) OR (Aerobic Trainings [Title/Abstract])) OR (Trainings, Aerobic [Title/Abstract])) OR (Aerobic Exercise Training [Title/Abstract])) OR (Aerobic Exercise Trainings [Title/Abstract])) OR (Exercise Training, Aerobic [Title/Abstract])) OR (Exercise Trainings, Aerobic [Title/Abstract])
#5 “Adolescent”[Mesh]
#6 (Adolescents [Title/Abstract])) OR (Adolescence [Title/Abstract])) OR (Teens [Title/Abstract])) OR (Teen [Title/Abstract])) OR (Teenagers [Title/Abstract])) OR (Teenager [Title/Abstract])) OR (Youth [Title/Abstract])) OR (Youths [Title/Abstract])) OR (Adolescents, Female [Title/Abstract])) OR (Adolescent, Female [Title/Abstract])) OR (Female Adolescent [Title/Abstract])) OR (Female Adolescents [Title/Abstract])) OR (Adolescents, Male [Title/Abstract])) OR (Adolescent, Male [Title/Abstract])) OR (Male Adolescent [Title/Abstract])) OR (Male Adolescents [Title/Abstract])
#7 #5 or #6
#8 “Child”[Mesh]
#9 (Children [Title/Abstract])
#10 #8 or #9
#11 #7 or #10
#12 #3 and #4 and #11

**Table 3:** Basic characteristics of the included studies.

Author Year	Country	Age (Year) (EG/CG)	Samples (EG/CG)	BMI (kg/m^2^) (EG/CG)	Intervention	Weight Status
Taketaka Hara·Hiroshi Fujiwara 2005 [25]	Japan	18.7 ± 1.3/ 18.4 ± 1.0	7/ 7	29.9 ± 1.8/ 33.5 ± 5.6	The AE group exercised 3 times a week for more than 30 min each time for a total of 8 weeks, with the exercise intensity set at the VT point (40.8~54.8% VO2max).	obesity
Aaron S. Kelly 2007 [26]	USA	10.8 ± 2.01/ 11.0 ± 2.25	9/ 10	32.7 ± 7.8/ 30.5 ± 7.3	The AE group exercised 4 times per week for a total of 8 weeks with progressively increasing intensity and duration of exercise. Participants began exercising at 50–60% of VO2max for 30 min each during the first week (including a 5-min warm-up and cool-down); halfway through the program, participants exercised at 60–70% of initial VO2max for 40 min; at the end of the program, participants exercised at 70–80% of initial VO2max for 50 min.	obesity
Eun Sung Kim 2007 [27]	Korea	17 ± 0.41/ 17 ± 0.38	14/ 12	29.6 ± 2.2/ 29.4 ± 2.4	The AE group of testers participated in supervised jumping for 40 min a day, 5 times a week for 6 weeks.	obesity
Michael Rosenbaum 2007 [28]	USA	13.7 ±0.7/ 13.6± 0.98	49/ 24	24.7 ± 9.8/ 24.3 ± 8.8	The AE group used dance and non-contact free sparring, exercising 3 times a week for a total intervention time of 3–4 months, with each exercise session lasting 45 min.	obesity
EMILY C-S MURPHY 2009 [29]	USA	7–12/ 7–12	23/ 12	27.9 ± 4.8/ 31.8 ± 5.0	Total duration of intervention for AE group 12 weeks, 3 times a week, 30 min each time.	obesity
Ji Young Kim 2011 [30]	Korea	17.63 ± 0.49/ 17.63 ± 0.49	18/ 12	28.79 ± 3.81/ 28.33 ± 2.84	The trial participants participated in 12 weeks of aerobic exercise for 50 min, 5 times per week, consisting of 10 min warm-up, 5 min cool-down and 35 min aerobic exercise. The aerobic exercise program (5 days/week) included running, stretching, rope skipping, badminton, basketball and aerobic dance.	obesity
Sung Soo Lee 2012 [31]	Korea	11.85 ± 0.38/ 12.62 ± 0.51	13/ 12	24.43 ± 1.47/ 24.26 ± 1.30	The AE group exercised on a treadmill 3 days a week at 50% HR for 12 weeks, until each exercise reached 300 kcal, monitored using a heart rate monitor, warmed up for 10–15 min and stretched for 10–15 min.	obesity
Min Liu 2018 [32]	China	14.6 ± 0.7/ 14.7 ± 0.8	30/ 20	33.9 ± 3.1/ 34.6 ± 4.9	AE group performed aerobic exercise for 4 weeks, 2 h each time with 5 min rest every 30 min, twice a day, 6 days a week, gradually increasing intensity from low (week 1 heart rate: 100–120 beats/min) to moderate (weeks 2–4 heart rate: 120–140 beats/min).	obesity

EG—experimental group; CG—control group; AE—aerobic exercise; VT—ventilatory threshold; HR—heart rate.

## Data Availability

Not applicable.
